# Rhinitis medicamentosa: a nationwide survey of Canadian otolaryngologists

**DOI:** 10.1186/s40463-019-0392-1

**Published:** 2019-12-09

**Authors:** James Fowler, Christopher J. Chin, Emad Massoud

**Affiliations:** 1 Dalhousie Medicine New Brunswick, Saint John, New Brunswick Canada; 2 Department of Surgery, Division of Otolaryngology – Head and Neck Surgery, Saint John, New Brunswick Canada; 3 Department of Surgery, Division of Otolaryngology – Head and Neck Surgery, Dalhousie University, Halifax, Nova Scotia Canada

**Keywords:** Otolaryngology, Rhinitis medicamentosa, Topical decongestant

## Abstract

**Background:**

Rhinitis medicamentosa is a non-allergic form of rhinitis that is typically caused by prolonged use of topical nasal decongestants. This condition commonly affects young adults and treatment is not trivial. We aimed to survey Canadian Otolaryngologists to determine practice patterns and their opinions regarding this under-studied condition.

**Methods:**

An electronic survey was sent to practicing Otolaryngologists within the Canadian Society of Otolaryngology – Head and Neck Surgery. The survey contained 16 questions pertaining to the diagnosis and treatment of rhinitis medicamentosa, as well as opinions on public and primary care awareness of proper use of nasal decongestants.

**Results:**

The survey was distributed to 533 Otolaryngologists and 69 surveys were returned (response rate of 13%). Cessation and weaning of decongestant (96%), and intranasal steroids (94%) were the most common methods for treating RM. Intranasal saline rinses (55%) and oral steroids (25%) were also supported by some respondents. For those who recommended cessation/weaning, 61% also concurrently introduced an intranasal steroid during this process. The majority responded that current warnings on nasal decongestants were inadequate (75%), and were not visible enough (79%).

**Conclusions:**

Rhinitis medicamentosa is a common, and very preventable condition. Although the literature lacks a standardized approach to RM, our survey has shown that many Otolaryngologists diagnose and treat RM in a similar manner. Treatment tends to focus on decongestant cessation, often with concurrent introduction of intranasal steroids. It was felt the warning labels on the topical medications are not currently satisfactory.

## Background

Rhinitis medicamentosa (RM) is a non-allergic form of rhinitis that is caused by prolonged use of topical nasal decongestants [1]. It can be seen when a patient uses a topical decongestant in excess of 5 days straight. Patients typically present with nasal congestion without rhinorrhea, post nasal drip, or sneezing [2]. RM most commonly affects young adults and accounts for approximately 1–9% of visits to an Otolaryngologist [3, 4].

**Fig. 1:**
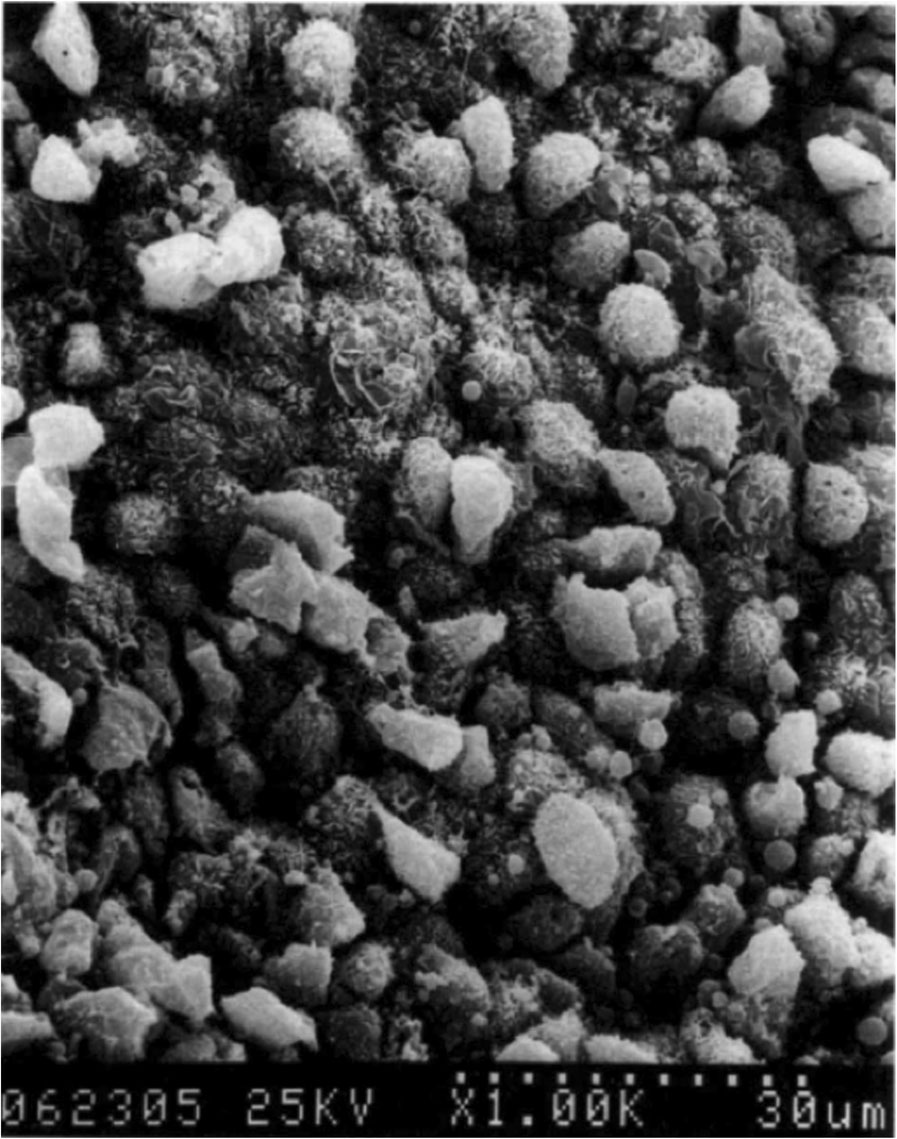
Electron microscopy of patient with rhinitis medicamentosa. Goblet cell hyperplasia, loss of cilia, and degenerative epithelial changes can be observed. Figure supplied by Dr. Sheen-Yie Fang, Department of Otolaryngology, National Cheng Kung University Hospital, Tainan, Taiwan

The pathophysiology of RM has been attributed to two classes of topical decongestants, sympathomimetic amines and imidazoline derivatives. Sympathomimetic amines act on alpha-1 and beta receptors; this is felt to result in a period of vasoconstriction followed by vasodilation, leading to nasal mucosa swelling [1–3]. Imidazoline is an alpha-2 agonist, which causes vasoconstriction of the nasal arteries/arterioles. This has a negative impact on endogenous norepinephrine. When the imidazoline is withdrawn, it is believed the sympathetic vasomotor tone cannot be maintained, which increases parasympathetic activity, and results in rebound congestion [1–3]. With time, chronic decongestant use leads to microscopic changes in the nasal mucosa resulting in goblet cell hyperplasia, squamous cell metaplasia, and destruction of the nasal cilia (Fig. 1) [5, 6].

Treatment and reversal of RM is not trivial. Currently, there is no formal standardization or established guidelines for treatment, other than avoidance of the topical decongestant [7]. Research has been conducted to analyze different treatment modalities and regiments, but the evidence is quite sparse. In general, results are based on animal models or studies with small sample size of healthy patients with study-induced RM [8]. The purpose of the present study is to survey Otolaryngologists within the Canadian Society of Otolaryngology – Head and Neck Surgery (CSOHNS) to better understand how RM is being diagnosed and treated in practice. Furthermore, the aim of the study is to identify practical solutions to increase patient safety, and potentially reduce the incidence of RM.

## Methods

Research Ethics Board approval was obtained (REB #2018–4620). A 16 question electronic survey (Additional file 1) was constructed in Opinio (ObjectPlanet Inc.). The survey was comprised of five sections: practice demographics, diagnosis and treatment, public awareness, and primary physician awareness. Each survey took approximately 5 min to complete electronically. An email was distributed to all registered Otolaryngologists within the CSOHNS, inviting them to participate in the study. Contained within the email was the research cover letter, as well as the online link to the survey. Participation was completely voluntary, and participants could decide to withdraw from the survey at any time. No personal or identifiable information was collected. All survey questions were reviewed by the CSOHNS Electronic Communication Chair.

Respondent data was exported from Opinio to Microsoft Excel (2019, Redmond, WA, USA). Descriptive statistics were used to analyze the data.

## Results

The survey was distributed to 533 Otolaryngologists registered to CSOHNS. Seventy-three responses were received, but four surveys were incomplete and were excluded.

### Practice demographics

Responses from all subspecialties of Otolaryngology – Head and Neck Surgery were captured within the survey. The practice demographics are shown in Table 1. The majority of the responses came from General Otolaryngology (49%), followed by the subspecialties of Rhinology (14%), and Head and Neck Oncology (12%). A slight majority of physicians reported working in a community practice setting (56%). Rhinitis medicamentosa was diagnosed and treated by all participating physicians. Fifty-two percent of Otolaryngologists recorded always screening for RM when evaluating a patient for nasal congestion or sinus issues. Overall, RM was infrequent with 48% of Otolaryngologists seeing “0-10 cases per year” and 33% seeing “11-20 cases per year”.

**Table 1:** Practice demographics of participating Otolaryngologists

Domains	Absolute Frequency	Relative Frequency
Years independent practice		
0–5 years	16	23.2%
6–10 years	17	24.6%
11–15 years	13	18.8%
16–20 years	4	5.8%
21–25 years	5	7.3%
Greater than 25 years	14	20.3%
Focus of practice		
General	52	49.0%
Rhinology	17	16.0%
Head and Neck Oncology	13	12.3%
Otology	6	5.7%
Pediatrics	8	7.6%
Facial Plastics	6	5.7%
Laryngology	3	2.8%
Other	1	0.9%
Practice setting		
Academic	17	24.3%
Community	39	55.7%
Mixed	14	20%
Cases of RM per year		
0–10 cases	35	50.7%
11–20 cases	24	34.8%
21–30 cases	5	7.3%
31–40 cases	3	4.4%
Greater than 50 cases	2	2.9%

### Diagnosis and treatment

Universally, a history of congestion and nasal decongestant use was diagnostic of RM (100%). Seventy-five percent of physicians also report using physical exam findings, including sinoscopy (sinonasal endoscopy) to aid in the diagnosis. The use of radiographic imaging was not common practice (1%).

Cessation and weaning of decongestant (96%), and intranasal steroids (94%) were the most common methods for treating RM. Intranasal saline rinses (55%) and oral steroids (25%) were also supported by some respondents. A minority of Otolaryngologists proposed the use of surgery (14%) and antihistamines (4%) to treat RM. With respect to weaning of the decongestant, 61% of surgeons described introducing an intranasal steroid during this process. Serial dilution of decongestant with saline (5%), dilution of decongestant while introducing an intranasal corticosteroid (14%), and stopping the decongestant “cold turkey” (8%) were less commonly used methods. The vast majority of respondents (75%) agreed that waiting for complete wean of decongestant was most appropriate when considering surgery for chronic rhinological disease.

### Public awareness

Opinions on visibility and adequacy of current nasal decongestant warnings were assessed. Seventy-nine percent of participants believed that warning labels were not visible enough, and 75% thought warnings were not adequate. For those who believed the warnings to be inadequate, they were also asked to make suggestions to improve safety. These responses can be divided into label specific, and pharmacy specific. Persistent themes, with respect to the product label, included: larger font, greater background-font contrast, larger label, “short-term use only – three days maximum”, and “this product is addictive”. Pharmacy specific changes included: restricting nasal decongestants to behind-the-counter, and requiring a prescription for the decongestant.

### Primary physician awareness

Lastly, respondents were asked about past inappropriate referrals, and opinions on nasal decongestant knowledge in the primary care setting. Fifty-nine percent report they have seen a patient in their practice who was actively encouraged to inappropriately use a nasal decongestant by another medical professional. Although this figure is high, the majority (61%) of respondents believe primary care physicians have adequate knowledge pertaining to the dangers of chronic decongestant use.

## Discussion

Rhinitis medicamentosa is a common, preventable condition that affects many patients seen in Otolaryngology – Head and Neck Surgery practices across Canada. The lack of standardized treatment protocols has made RM frustrating to treat.

A recent systematic review by Zucker et al. [7] suggests the literature currently lacks compelling evidence to formally construct a standardized treatment plan for RM. Although this may be true, there are three randomized control trials that support the immediate cessation of the decongestant, and simultaneous introduction of nasal steroids. The use of intranasal fluticasone propionate for the treatment of RM has been studied by both Hallen et al. [9] and Vaidyanathan et al. [10]. In their studies, intranasal fluticasone propionate was administered daily for 14 days and 3 days, respectively. Over the short study durations, patients who were given intranasal fluticasone propionate reported rapid resolution of their nasal symptoms, and objectively, had significantly reduced nasal swelling. Similar findings were seen in a study conducted by Ferguson et al. [11], who investigated the effectiveness of daily intranasal budesonide. In their study, the treatment group administered budesonide over a 2 week period, following 4 weeks of 0.05% oxymetazoline. In their study, 20 patients with allergic rhinitis were randomly assigned to intranasal budesonide or placebo groups. All subjects halted the use nasal decongestants for the first week, and then were initiated on 2 sprays of 0.05% oxymetazoline twice a day. During week four, patients were started on intranasal budesonide or placebo spray, and in week five the decongestant was stopped. For the budesonide group, nasal volume and minimal cross-sectional area were significantly increased upon initiation of steroid therapy, compared to placebo. Rebound congestion remained controlled during the observation period for the treatment group.

These three studies show the potential of intranasal corticosteroids for treatment of RM. Interestingly, it is frequently taught that intranasal steroids should be used for closer to 6–8 weeks to see optimal effect, suggesting their studies may not have seen the medication when it is at maximal effectiveness. The responses of our survey are in keeping with the literature. Respondents showed a strong consensus for cessation of the decongestant and initiation of intranasal steroids. Weaning of decongestants, while introducing intranasal steroids to provide relief during the withdrawal period, was supported by the majority of respondents (61%).

Fourteen percent of our survey participants reported using surgical intervention in the treatment of RM. Caffier et al. [12] conducted a prospective clinical trial investigating the outcomes of diode laser inferior turbinate reduction in patients with chronic, refractory RM. Forty-two patients were involved, and were followed 12 months post-intervention. Mean decongestant addiction time was 5 ± 2 years. Post-operative results revealed significant improvement in subjective and objective nasal airflow. Eighty-eight percent of participants were able to completely stop nasal decongestant use by the six-month follow-up. This limited evidence suggests there may be a role for surgery but more rigorous study needs to be done before recommendations can be made.

Despite respondent support for other treatment regimens, such as saline rinses, oral steroids, and antihistamines there is little backing for their use within the literature. A recent review [2] outlined the evidence for each of the aforementioned treatments. The level of evidence is weak, as no randomized control trials have been completed, and conclusions are based solely on case reports and case series. No studies could be found to support serial weaning, or abrupt cessation of decongestants.

One of the reasons this study was initiated was the consistent observation that many patients we saw were seemingly oblivious to the dangers of prolonged use of topical decongestants. We felt that a major contributor to this was a lack of public knowledge, due to poor warning labels, and easy access to decongestants. The majority of our respondents thought the labels lacked visibility (79%), and were inadequate in conveying dangers associated with the medication (75%). Warning labels are critical for patient safety, but unfortunately, many are poorly placed, small in size, and lack contrast with other information [13]; this may be intentional on the part of the pharmaceutical company. As well, evidence suggests that although most consumers are aware of product labels (88% in 1 study), less than half will fully read them (46% in the same study), and even fewer (27%) will retain the information [14]. Our respondents were asked about ways they felt that the labels could be altered, and many physicians felt that the warnings should be in larger font, more easily seen, contrast the background, and be explicit (for example: “This product is very addictive and should not be used more than 4 days in a row” or “Using this medication for more than 3 consecutive days will worsen nasal congestion”). It was also suggested by multiple respondents that these medications should be moved “behind the counter”, requiring a request to a pharmacist to obtain them, or even requiring a prescription.

Our study has some significant limitations. The study design was a survey, which, while useful for gaining an understanding of what our colleagues are doing to treat RM, does not tell us if these treatments are actually effective. As well, our response rate was 13%, which is similar to other surveys within the CSOHNS, but would be considered a limitation to our study.

In general, RM is an understudied condition which lacks a consensus on treatment even though every respondent to the survey treats this condition. Future avenues of study could focus on the efficacy of the various treatment modalities, as well analyze the effects of reducing access to the medications and/or increasing the visibility of the warning labels.

## Conclusions

Rhinitis medicamentosa is a common, and preventable condition. Although the literature lacks a standardized approach to RM, our survey has shown that many Otolaryngologists generally treat RM in a similar manner. Respondents typically treat RM by encouraging decongestant cessation, with concurrent introduction of intranasal steroids. Respondents felt that the warning labels on the medications are not currently adequate/visible to prevent the condition.

## Supplementary information

**Additional file 1.** Survey Questionnaire.

## Data Availability

All data generated or analysed during this study are included in this published article.

## Abbreviations

CSOHNS: Canadian Society of Head and Neck Surgery
RM: Rhinitis medicamentosa
