# Supplementary material for: Rhinitis medicamentosa: a nationwide survey of Canadian otolaryngologists
Source: J Otolaryngol Head Neck Surg. 2019 Dec 9;48:70. doi: 10.1186/s40463-019-0392-1 (PMC6902618; doi:10.1186/s40463-019-0392-1)
Supplement: Supplementary file 1 — Additional file 1. Survey Questionnaire. [file 40463_2019_392_MOESM1_ESM.docx]

**Survey Questionnaire:**

**Profile of Otolaryngology Practice:**

1. How long have you been in independent practice (post-residency/fellowship)?
   1. 0-5 years
   2. 6-10 years
   3. 11-15 years
   4. 16-20 years
   5. 21-25 years
   6. More than 25 years
2. How would you describe the focus of your practice (choose all that apply)?
   1. General
   2. Rhinology
   3. Head and Neck Oncology
   4. Otology
   5. Pediatric
   6. Facial Plastics
   7. Laryngology
   8. Other: _________________
3. How would you describe your practice setting?
   1. Academic
   2. Community
   3. Both
   4. Other: _________________
4. Have you ever treated a patient with Rhinitis Medicamentosa (RM) in your practice?
   1. Yes
   2. No
5. When you receive a referral for nasal congestion or sinus issues, do you typically screen for Rhinitis Medicamentosa?
   1. Always
   2. Most of the time
   3. Sometimes
   4. Rarely
   5. Never
6. On average, how many cases of Rhinitis Medicamentosa do you see in your practice per year?
   1. 0-10 cases
   2. 11-20 cases
   3. 21-30 cases
   4. 31-40 cases
   5. 41-50 cases
   6. Greater than 50 cases

**Diagnosis:**

1. When diagnosing RM, the diagnosis is made using (select all that apply):

- History of congestion and topical decongestant usage
- Physical examination (including sinoscopy)
- Imaging
- Other (i.e. nasal rhinometry): _________________

**Treatment:**

1. How do you typically treat Rhinitis Medicamentosa (select all that apply)?
   1. Oral steroids
   2. Nasal steroids
   3. Saline
   4. Surgery
   5. Antihistamine
   6. Cessation/weaning of topical decongestant
   7. Other: _________________
2. If you recommend cessation/ weaning, what do you typically recommend to a patient who is actively using a nasal decongestant?
   1. ‘Wean off’ the decongestant on their own
   2. “Wean off” the decongestant while introducing intranasal steroid preparation
   3. Serially dilate the decongestant with saline over time
   4. Serially dilute the decongestant while introducing intranasal steroid preparation
   5. Stop the decongestant ‘cold turkey’
   6. Other: _________________
3. If there is ongoing chronic rhinosinusitis or deviated nasal septum, would you wait until the patient is off the decongestant to perform the surgery?
   1. Yes, I would wait
   2. No, I would perform the surgery while the patient is using the decongestant

**Public Awareness:**

1. Do you feel that the current warnings on decongestant medications are adequate?
   1. Yes, they are adequate
   2. No, they are not adequate
2. Do you feel that the current warnings on decongestant medications are visible enough?
   1. Yes, they are visible
   2. No, they are not visible enough
3. If you feel the current warnings are inadequate, what more would you suggest the warning should include?

__________________________________

__________________________________

__________________________________

**Primary Care Physician Awareness:**

1. In your practice, have you seen a patient who has been actively encouraged to use a topical decongestant from another medical professional?
   1. Yes
   2. No
2. Do you feel that primary care physicians have adequate knowledge of the risks associated with chronic nasal decongestant use?

a) Yes

b) No

16. How do you think awareness can be increased at the primary care level?

- 1. Publish a statement within newsletter by College of Family Physicians of Canada
  2. Official statement made by the Canadian Society of Otolaryngology - Head and Neck Surgery.
  3. Provide educational workshops at College of Family Physicians of Canada Annual General Meeting
  4. Other: _________________
